# Neural Circuit Connections and Functions of Locus Coeruleus–Norepinephrine System

**DOI:** 10.3390/ijms262211163

**Published:** 2025-11-19

**Authors:** Mei Hao, Fang Li, Jia-Wen Duan, Ming-Hu Han

**Affiliations:** 1 The Brain Cognition and Brain Disease Institute, Shenzhen Institute of Advanced Technology (SIAT), Chinese Academy of Sciences, Shenzhen 518055, China; m.hao@siat.ac.cn (M.H.); f.li@siat.ac.cn (F.L.); 2 Department of Mental Health and Public Health, Faculty of Life and Health Sciences, Shenzhen University of Advanced Technology (SUAT), Shenzhen 518107, China; duanjiawen@suat-sz.edu.cn; 3 Department of Pharmacological Sciences, Friedman Brain Institute, Icahn School of Medicine at Mount Sinai, New York, NY 10029, USA

**Keywords:** locus coeruleus, norepinephrine, evolutionary feature, neural circuit, neuropsychiatric disorders

## Abstract

The locus coeruleus-norepinephrine (LC-NE) system is a phylogenetically conserved neuromodulatory hub that regulates fundamental brain states and behaviors, including arousal, cognition, emotion, and pain. This review integrates two critical perspectives to provide a unified framework for understanding this system. First, we synthesize the evolutionary trajectory of the LC from non-mammalian to mammalian vertebrates, highlighting conserved properties and changes in cell number, anatomical projections, and physiological functions. Second, we detail the intricate connectivity of its afferent and efferent circuits, explaining how specific inputs and outputs modulate LC activity and govern diverse behaviors under physiological and disease conditions. Together, we aim to highlight the central role of the LC in brain function and disease through an evolutionary and circuit-based lens.

## 1. Evolution of Norepinephrine System in Locus Coeruleus

### 1.1. Evolutionary Conservation of the Locus Coeruleus

The locus coeruleus (LC) is a vertebrate-specific nucleus, serving as a crucial regulatory hub across various vertebrate species. Despite its diminutive size, the LC exerts a profound influence through its extensive axonal projections, releasing norepinephrine (also known as noradrenaline, NE) to modulate brain states and behaviors [1,2,3]. The LC serves as a conserved neuromodulator system across vertebrates. For example, in zebrafish, it coordinately modulates brain states and promotes wakefulness, and in mammals, it is pivotal for promoting arousal, highlighting its evolutionary conservation [4,5]. In Section 1, we delve into the evolutionary trajectory of the LC from non-mammalian to mammalian vertebrates, highlighting its morphological changes, anatomical projections, and physiological functions. Moreover, we also explore sex differences in LC-NE system in mammalian vertebrates, shedding light on structural and genetic disparities that contribute to divergent behavioral responses.

### 1.2. Evolutionary Features of the LC from Non-Mammalian to Mammalian Vertebrates

#### 1.2.1. Number of Cells

**Figure 1:**
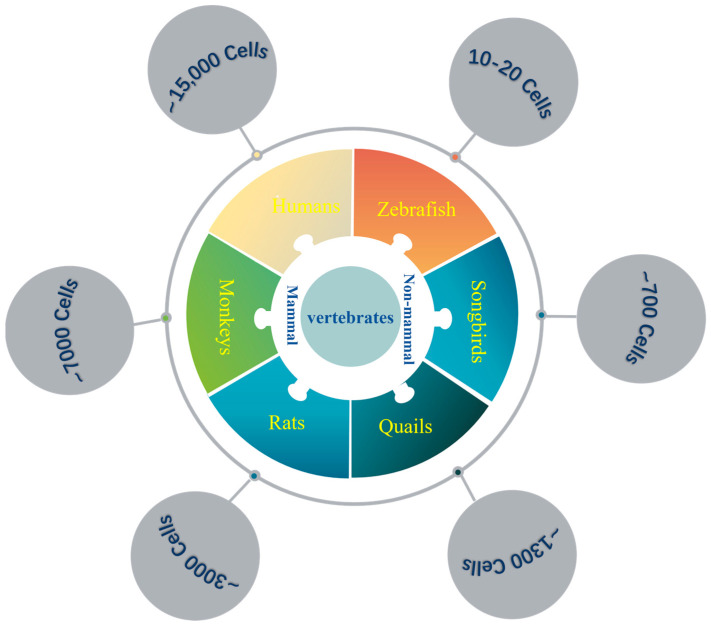
**The number of LC neurons in different species.** The left side shows the number of mammalian LC cells: rats have ~3000 cells, monkeys have ~7000 cells, and humans have ~15,000 cells. The right side shows the number of non-mammalian LC cells: zebrafish have ~10–20 cells, songbirds have ~700 cells, and quails have ~1300 cells.

To exert control over a broader range of brain regions and support increasingly complex physiological functions and behaviors, the number of neurons within the LC undergoes a gradual increase along the evolutionary transition from non-mammalian to mammalian vertebrates. In the case of zebrafish, an ancient vertebrate lineage that emerged ~340 million years ago, the LC comprises only 10–20 cells [6,7]. Along the non-mammalian evolutionary path, this number escalates in avian species such as songbirds and quails, with ~700 and ~1300 LC neurons, respectively [8]. Conversely, rats, as one of the most extensively studied experimental models among mammalian organisms, harbor ~3000 LC neurons, which are more than double that of quails. Moving along the mammalian evolutionary trajectory, the number of LC neurons in monkeys demonstrates a substantiated rise to ~7000 neurons [9]. Humans, on the other hand, with the most sophisticated brain structure, exhibit a staggering increase in LC neurons to ~15,000 cells, which are involved in regulating various kinds of behaviors and physiological functions [9,10] (Figure 1). Interestingly, despite this increase in neuron number, the proportion of LC neurons relative to the total number of brain neurons gradually decreases across evolution (except for avian species), highlighting the scaling of the LC neurons within an enlarging brain [11,12] (Table 1).

#### 1.2.2. Anatomical Projections

Although the LC contains a relatively low number of cells compared to other brain regions, its broad axonal projections enable it to modulate nearly the entire brain in both non-mammalian and mammalian vertebrates. At the neural circuit level, the LC also demonstrates evolutionarily conserved features, with both non-mammalian vertebrates and mammalian vertebrates possessing conserved ascending and descending pathways. Ascending pathways extend to the telencephalon, diencephalon, and mesencephalon, while descending pathways project to the rhombencephalon and the spinal cord (SC) [13,14]. In fish, the LC axonal project to the telencephalon, optic tectum, hippocampus (HIP), cerebellum (CB), and SC [15,16]. Comparatively, amphibians and reptiles, apart from sharing identical brain regions, exhibit an additional array of projection regions, including the dorsal medial cortex of the telencephalon and the substantia nigra of the mesencephalon [17,18,19,20]. Birds, on the other hand, possess unique projection sites absent in amphibians and reptiles, namely, the wulst and caudomedial nidopallium, which are associated with specialized physiological functions such as the production of distinctive birdsongs [21,22,23,24]. In mammals, LC neurons project to amygdala (AMY) and HIP in the diencephalon, as well as the ventral tegmental area (VTA) in the mesencephalon, indicating their functional role of LC in the regulation of emotions, learning, and memory [25,26].

#### 1.2.3. Physiological Functions

Plentiful studies have substantiated that the LC is a key node in regulating arousal across both non-mammalian and mammalian vertebrates. In larval zebrafish, the LC promotes the transition from anesthesia to alertness [27]. A recent study revealed that zebrafish are induced into anesthesia state quickly and exhibit a slowly gradual recovery from the anesthesia state when LC neurons are locally lesioned or NE is depleted [27]. Similarly, in mice, optogenetic and pharmacological activation of LC neurons results in an immediate switch from the state of sleep to wakefulness, while inhibition of LC neurons reduces the duration of wakefulness [28,29].

The LC also plays a crucial role in regulating sensorimotor transformations in both non-mammalian and mammalian vertebrates. In songbird, a commonly used model for exploring sensorimotor transformation, the caudomedial nidopallium—analogous to the mammalian secondary auditory cortex—receives NE inputs from the LC [29]. Experimentally manipulating NE signaling in this region yields distinct behavioral outcomes: for instance, upregulating NE signal increases the ratio of signal-to-noise, while downregulating NE signal disrupts the selectivity of birdsongs [30]. In mammals, LC activity similarly influences sensorimotor function. Utilizing electrical stimulation method, study in rats has shown that stimulation of the LC suppresses trigeminal sensorimotor functions in rats [31]. Specific behaviors such as the nociceptive flexion reflex in limbs [32], the tail flick reflex [33], and the vocalization response to paw pressure [34] are all suppressed by the activation of LC neurons.

### 1.3. Sex Differences in the LC of Mammalian Vertebrates

Sex differences represent an interesting and important aspect of understanding the evolutionary trajectory of the LC. Structural investigations have found that females rats possess a greater number of NE neurons in the LC core regions and longer dendrites in peri-LC regions, which increase the likelihood of LC neurons connecting with afferents [35,36]. The sex difference in the size of the LC varies across rat strains. For example, the sexual dimorphism observed in Long-Evans rats is absent in the ancestral Wistar strain [37,38]. Human studies have further contributed to our understanding, reporting ~18,300 neurons in women and ~15,700 neurons in men within the LC, further illustrating sex differences in humans as well [39,40]. Moreover, a recent study identified more than 3000 genes expressed in the LC, with over 100 of these genes presenting significant sex differences, and this study also proved that those >100 genes contribute to sex-related behavioral responses [41]. Even though both sexes share broad similarity in input patterns, the input proportions differ quantitatively. In males, the afferent inputs arise primarily from the interbrain, and cerebrum, whereas in female, the afferent inputs predominantly originate from the midbrain and hindbrain [42].

## 2. Efferent and Functions of NE Neurons in the LC

**Figure 2:**
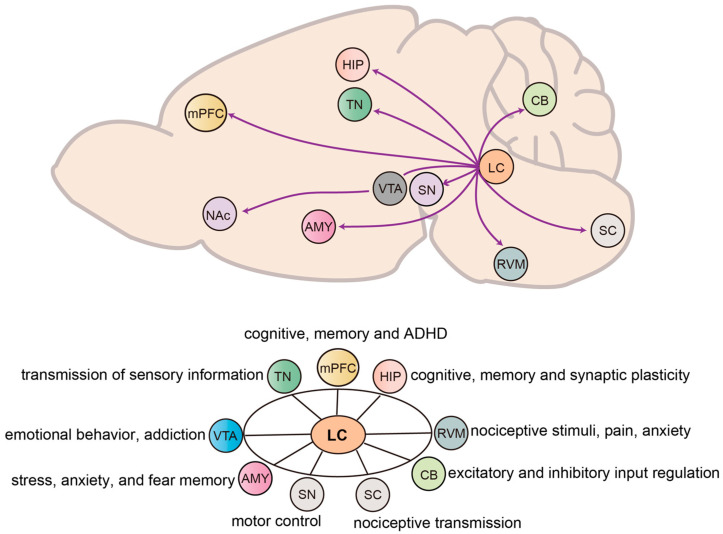
**Projection circuit and functional target of the LC in mice.** The LC sends widespread noradrenergic projections to multiple brain regions, including mPFC, hippocampus (HIP), amygdala (AMY), thalamic nuclei (TN), ventral tegmental area (VTA), nucleus accumbens (NAc), rostral ventromedial medulla (RVM), substantia nigra (SN), cerebellum (CB) and spinal cord (SC). These projections contribute to diverse functions such as cognitive processing, emotional regulation, sensory modulation, motor coordination, and pain perception.

The LC serves as the primary source of NE in the brain, with its efferent axons projecting widely throughout the entire forebrain, brainstem, CB, and SC [43]. This extensive projection network suggests that the LC plays an important role in brain functions and the regulation of behavior through its participation in diverse neural circuits (Figure 2). In this section, we provide a detailed overview of the efferent targets of LC neurons, organized in major brain regions: (1) forebrain, including the medial prefrontal cortex (mPFC), HIP, AMY, and thalamus; (2) midbrain, encompassing the VTA and substantia nigra; (3) hindbrain, covering structures such as the CB and brainstem nuclei; and (4) SC, highlighting LC contributions as a central regulator to diverse physiological and behavioral processes, with broad relevance to health and disease.

### 2.1. Forebrain

#### 2.1.1. LC to Medial Prefrontal Cortex

The mPFC, a crucial brain region associated with higher cognition and emotional regulations, serves as a primary target for the LC projections. A substantial body of evidence indicates that the LC serve as a major source of NE for the mPFC [44]. Dysregulation of the LC-mPFC NE system has been implicated in multiple neurological and psychiatric disorders, including memory impairments, attention-deficit/hyperactivity disorders (ADHD) [45], and stress-related disorders [46]. Studies have shown that increased NE release in the LC-mPFC NE system enhances working memory and cognitive flexibility [47], and is particularly important for mPFC engram early tagging and the storage of remote fear memory [48]. Disruption of β1-adrenergic receptor (β1-AR), which are primarily activated by epinephrine and NE, in the mPFC leads to impairment of the storage of remote fear memory, while enhancement of β1-AR signaling promotes engram early tagging and storage of remote memory in juvenile mice [48]. In addition, NE in the mPFC is also essential for the regulation of attention. Reduced NE afferents to the mPFC impair attention shifting, while increasing noradrenergic neurotransmission in the mPFC, acting on the α1-adenergic receptors in rats, enhances performance on attention switching task [49,50] and improves sustained attention induced by the psychostimulant methylphenidate [51].

In contrast, in pain model, activation of the LC-mPFC circuit increases spontaneous pain as well as induces negative affective responses such as aversion and anxiety-like behaviors [52]. Long-term chronic stress enhances NE innervation while reducing dendritic spines in mPFC pyramidal cells, contributing to stress-related cognitive dysfunction [53]. It is observed that both excessive and insufficient NE transmission in the mPFC can lead to behavioral disturbances, which again highlights the vital role of LC-mPFC NE signaling in regulation of central nervous system’s functions and behaviors.

#### 2.1.2. LC to Hippocampus

The HIP is an important brain region associated with learning, memory, and cognition. Previous studies have consistently shown that the LC is the primary source of HIP NE [54]. NE released from the LC has been shown to be involved in multiple phases of HIP-dependent memory processing, including encoding, consolidation, retrieval, and reversal [25,55]. Early lesion and stimulation studies provide strong evidence for this role of the LC. Bilateral or unilateral LC lesions, or immunotoxic ablation all impair spatial memory in spatial and working memory [56,57]. Conversely, electrical stimulation of the LC leads to NE released in the rodent hippocampal dentate gyrus, enhancing the encoding of special memory and facilitating memory retrieval via β-adrenergic receptor [58,59,60]. These findings highlight the critical role of the LC-HIP pathway in regulating memory.

At the molecular and synaptic level, the LC regulates memory storage in HIP (particularly in the dentate gyrus) via the corelease of NE and dopamine, facilitating HIP long-term plasticity in the forms of long-term depression (LTD) and long-term potentiation (LTP) via both DA D1/D5 receptors and β-adrenergic receptors [61,62]. However, the interaction between NE and dopamine in LC-related HIP-dependent memory remains to be elucidated. Previous study has indicated that activation of LC NE neurons in rodents enhances spatial memory through D1/D5 receptors but not β-adrenergic receptors [63]. A recent study has shown that increasing NE release in the HIP facilitates the contextual associative learning through β-adrenergic receptors and can rescue deficits caused by dopaminergic dysregulation in the HIP [64].

In vitro experiments in rats have further indicated that β-adrenergic receptor agonist can facilitate HIP sharp wave ripples, the oscillatory patterns produced by synchronous neural events, which facilitate memory consolidation [65]. Beyond local HIP circuits, LC activation has also been found to enhance HIP–PFC LTP, exerting a significant influence on higher-order cognitive functions [66]. Importantly, recent research indicates that impaired HIP synaptic plasticity associated with Alzheimer’s disease is closely linked to the degeneration of the LC. This degeneration primarily manifests as dysregulation of noradrenergic and dopaminergic release in both the LC and HIP, resulting in impaired memory [67]. Together, these findings highlight the LC as a critical regulator of HIP synaptic plasticity and memory, with its function and dysfunction directly contributing to memory processing.

#### 2.1.3. LC to Amygdala

The AMY, a subcortical structure located in the temporal lobes of the brain, is widely recognized for its involvement in the onset of stress, anxiety, fear, addiction, and epilepsy, functioning as an important brain region associated with emotions. The LC emits a wide range of noradrenergic projections to different subregions of the AMY, which are critical for stress, anxiety, and fear memory processing [68]. Activation of the LC-basolateral amygdala (BLA) pathway has been found to increase NE release in the BLA and alter BLA neuronal activity via β-adrenergic receptors, which preferentially increase the activity of BLA neurons that project to areas known to modulate negative affect, thereby promote fear and aversive learning and amplify anxiety-like behaviors [69,70,71]. The LC-central amygdala lateral division (CeL) circuit plays a crucial role in the retrieval of conditioned context-induced morphine withdrawal memory, which is mainly manifested by the binding of NE to α1-adrenoreceptors in the CeL in synergy with glutamatergic receptor phosphorylation [72]. A recent study has further implicated the LC-AMY pathway in stress response, showing that rats with memories of severe trauma that were resistant to reconsolidation therapy with anisomycin become responsive when the NE signaling from the LC is blocked, providing a potential therapeutic target for post-traumatic stress disorder (PTSD) [73].

Moreover, LC-released NE in AMY plays a significant modulatory role in amygdaloid kindling seizures, with activation of LC reducing susceptibility to amygdaloid kindling seizures. Overall, these findings highlight the LC-AMY pathway as one of the central regulators of emotional memory and stress-related behaviors, with its dysregulation contributing to both affective disorders and seizure susceptibility.

#### 2.1.4. LC to Thalamic Nuclei

The lateral geniculate nucleus (LGN), anatomically situated within the posterior portion of the thalamus, receives projections from NE-containing neurons in the LC and plays a crucial role in the transmission of sensory information [74,75,76]. Through these projections, activation of the LC can increase the binding of NE to α1-adrenoceptors in the LGN, consequently amplifying its responsiveness to afferent stimuli, whereas α1-adrenoceptor antagonists attenuate this effect [75]. The enhanced LGN responsiveness can be obtained following long-term use of tricyclic antidepressants, which involve α1-adrenergic receptors, indicating that this modulatory effect may have clinical relevance [77]. Furthermore, unilateral injury to the visual cortex has been shown to increase the projection of LC NE neurons to the LGN, which are primarily mediated through β-adrenergic receptors, suggesting a potential compensatory mechanism to maintain sensory processing.

Beyond the LGN, the paraventricular thalamus (PVT) is another key LC-targeted subregion within the thalamus, serving as a critical node for stress responses and arousal regulation [78,79]. It receives dense tyrosine hydroxylase (TH)-positive inputs from the LC [80,81], enabling LC-mediated modulation of arousal. Research has found that optogenetic activation of LC-PVT projections facilitates arousal from isoflurane anesthesia, suggesting a potential therapeutic strategy for improving recovery following general anesthesia [80]. Furthermore, stress-induced disinhibition in the posterior PVT is mediated by LC-driven elevation of extracellular dopamine in the midline thalamus. This process depends on dopamine D2 receptor activity in PVT neurons and results in heightened stress sensitivity, emphasizing the LC’s role in mediating stress-related PVT signals [81]. Overall, LC projections to thalamic nuclei contribute to both sensory processing and the regulation of stress and arousal, underscoring their integral role in adaptive brain function.

### 2.2. Midbrain

#### 2.2.1. LC to Ventral Tegmental Area

The VTA, located in the midbrain, is a primary region responsible for dopamine release and is integral to two major dopamine neural pathways: the mesolimbic and mesocortical pathways [82]. As one of the major downstream regions of the LC, the LC-VTA pathway is closely related to the regulation of emotional behaviors [83,84]. NE released by the LC not only increases the excitability of dopamine cells in the VTA, but also exerts inhibitory effects on dopamine cells activity in reverse [85,86]. In a mouse model of chronic social defeat stress-induced depression, the activity of the VTA-nucleus accumbens (NAc) dopamine system was elevated, and activation of the LC-VTA NE pathway reduced this hyperactivation of the VTA-NAc dopamine system and reversed depression-like behaviors in susceptible mice, primarily via α1- and β3- adrenergic receptor signaling in the VTA [84]. These findings are consistent with previous reports indicating that NE release in the VTA suppresses the activity of dopamine cells [87].

Additionally, given that LC NE regulates the VTA dopamine neurons excitability, the LC-VTA pathway is also intricately involved in the regulation of addiction [88]. Recent study has confirmed the significant role of the LC-VTA pathway in cocaine seeking behaviors, finding that optogenetic inhibition of LC NE terminals in the VTA attenuates seeking behavior throughout the cocaine seeking session, whereas it increases seeking behaviors when inhibition is delivered contingently upon an active lever press [89]. Together, these findings highlight the LC-VTA pathway plays a crucial role in regulating dopaminergic activity, influencing emotion-related behaviors and drug addiction.

#### 2.2.2. LC to Substantia Nigra

The substantia nigra (SN), a midbrain structure within the basal ganglia, plays a critical region for dopamine production, which is essential for regulating motor control and reward-related behaviors [90]. Recent studies have shown that LC NE neurons send complex axonal projections to the dopaminergic neurons in the SN [90]. Degeneration of these dopaminergic neurons in the SN is a well-established pathological hallmark of Parkison’s disease (PD) [91]. Notably, clinical observations indicate a spatially extensive degeneration of LC neurons in PD patients, with neuronal loss occurring throughout the extent of the LC [92,93]. This sequence of degeneration suggests that LC pathology may precede SN degeneration, positioning LC pathology as a potential early biomarker for the preclinical detection of PD. Study has provided potential evidence for this assumption, demonstrating that the LC neurodegeneration and Lewy bodies accumulation occur prior to the onset of PD pathological processes in the SN [94]. Recent published study has further heightened the role of LC-SN connection in PD, demonstrating the protective role of LC NE neurons against dopaminergic neuronal depletion in the SN. In mice, overexpressing human α-synuclein A53T missense mutation enhanced activity of LC NE neurons, mitigated dopaminergic neuron loss and prevented motor deficits [90], underscoring the potential therapeutic relevance of the LC-SN pathway in PD pathophysiology.

### 2.3. Hindbrain

#### 2.3.1. LC to Rostral Ventromedial Medulla

A substantial body of studies have demonstrated that LC emits noradrenergic projections to both the brain and SC, with spinal projections playing a key role in regulating analgesic function [46,95]. The principal mechanism of this regulation revolves around the control of NE in the spinal dorsal horn [96]. Within the descending endogenous pain modulation system, the rostral ventromedial medulla (RVM) serves as a critical hub for pain regulation [97,98]. Its downward projection to the spinal dorsal horn modulates the transmission of nociceptive stimuli, exerting either facilitatory or inhibitory effects depending on the context [99]. As both the LC and RVM are core elements of the pain modulation circuitry, they share a common pathway that co-regulates the response to noxious stimuli [100]. Recent studies have found that noradrenergic neurons from the LC, mainly located in the dorsal caudal LC, project to RVM, and the dysfunction of this LC-RVM pathway is associated with the mis-transmission of injury-related information and stress-induced disorders [99]. Chemogenetic activation of the LC-RVM pathway has been shown to promote visceral hyperalgesia as well as stress-induced anxiety-like behaviors in mice, effects primarily mediated via α1-adrenoceptors within RVM [99]. Together, these findings highlight the LC–RVM pathway as a critical regulator of descending pain modulation, with its dysfunction contributing to hyperalgesia and stress-related behavioral alterations.

#### 2.3.2. LC to Cerebellum

The LC projects noradrenergic fibers to the CB, releasing NE onto cerebellar Purkinje cells [101]. NE has been found to modulate the induced response of Purkinje cells to both excitatory and inhibitory inputs. Notably, NE released from LC terminals has been widely studies for its ability to enhance the responsiveness of γ-aminobutyric acid (GABA) receptors in Purkinje cells, thereby enhancing inhibitory signaling [102]. The main mechanism of this effect has been shown to occur primarily through NE binding to β1-ARs on Purkinje cells, leading to increased intracellular cyclic adenosine monophosphate (cAMP) levels through a series of reactions and ultimately enhancing the action of GABA [103]. Additionally, NE released from LC terminals also affects the input response of Purkinje cells to climbing fiber, with increased NE release enhancing the excitatory input of climbing fiber induced by Purkinje cells [104]. In addition, the release of NE also affects the efficacy of basket and stellate cell on Purkinje cells, with a more pronounced effect observed in young rats compared to old ones [105]. Recent research revealed that NE released from the LC to the CB is involved in aversive learning. Chemogenetic and optogenetic inhibition of the LC-CB pathway blocks the formation of fear memory without affecting motor function in mice [106]. Taken together, these observations suggest the LC–CB pathway as a critical modulator of Purkinje cell activity and inhibitory-excitatory balance, with important implications for both motor control and aversive learning.

### 2.4. Spinal Cord

#### LC to Spinal Cord

The LC sends noradrenergic fibers to the spinal dorsal horn and mainly targets to its superficial layer, which projection constitutes a primary source of NE in the spinal dorsal horn and plays an important role in pain modulation [107]. Extensive studies have highlighted the association between the LC-SC pathway in the descending modulation of nociceptive transmission. When subjected to nociceptive stimuli, the LC releases increased amount of NE, which, upon combing with adrenoceptors in the SC, inhibits the transmission of nociceptive information [108,109]. Recent research has uncovered that reducing neuroinflammation in astrocytes and microglia within the spinal dorsal horn is also one component of the analgesic effect mediated by the LC-SC pathway [109]. It should be noted that while LC NE neurons have been found to elicit analgesic effect via the LC-SC pathway, they also provide substantial amount of NE to the mPFC, which, as we discussed above, can exacerbate pain and anxiety-like behaviors. Although the results may appear contradictory, a recent study has discovered that these two noradrenergic neuronal populations within the LC operate independently, providing evidence for a modular functional organization of the LC [46]. Furthermore, different subpopulations within the LC through the SC exhibit bidirectional responses to thermal nociception, with antinociception effect originating from neurons in the ventral region, likely mediated via projections to the dorsal horn of the SC [95,110]. All together, these findings further emphasize the essential functional role of LC-SC pathway in the context of pain modulation, with distinct LC subpopulations exerting independent yet complementary roles in the regulation of analgesia and affective pain responses.

## 3. Inputs and Functions of NE Neurons in the LC

**Figure 3:**
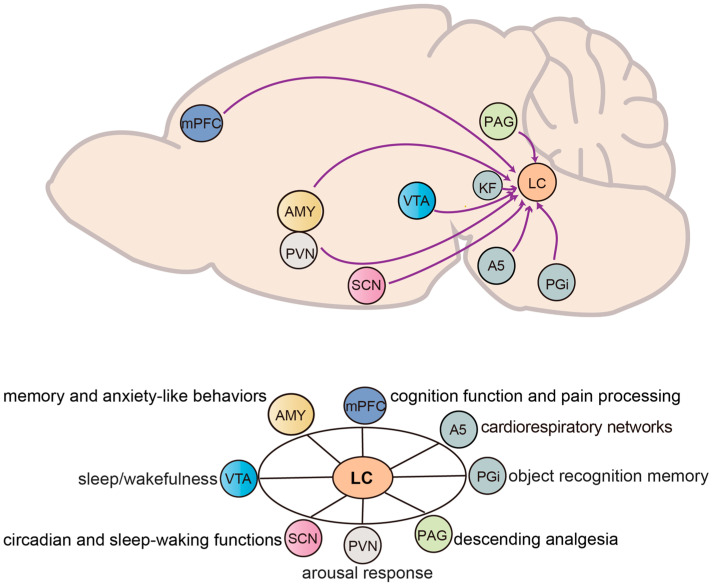
**Inputs and functions of the LC in mice.** The LC receives afferent projections from several brain regions, including the nucleus paragigantocellularis lateralis (PGi), ventral tegmental area (VTA), suprachiasmatic nucleus (SCN), midbrain periaqueductal gray (PAG), amygdala (AMY), medial prefrontal cortex (mPFC), paraventricular nucleus (PVN), Dorsomedial hypothalamic nucleus and perifornical area (DMH/PeF), parabrachial nucleus (PB), Kölliker-Fuse nucleus (KF). These inputs modulate diverse LC-mediated functions such as arousal, circadian rhythm regulation, stress response, pain modulation, cognitive processing, and cardiorespiratory networks.

The LC is regulated by a wide array of afferent inputs that shape its activity and, in turn, its influence on target circuits and brain regions. These inputs arise from both subcortical and cortical structures, including the brainstem, midbrain, hypothalamus, forebrain, and pontine, each providing excitatory, inhibitory, or modulatory control over LC neurons. Through this diverse afferent network, the LC integrates signals related to arousal, stress, pain, emotion, circadian rhythms, and autonomic states, allowing it to adjust NE output according to behavioral and physiological demands. In the following subsections, we review major input pathways to the LC, emphasizing their functional contributions and roles in shaping state-dependent LC activity (Figure 3).

### 3.1. Midbrain and Brainstem

#### 3.1.1. Paragigantocellularis to LC

The nucleus paragigantocellularis lateralis (PGi), situated in the rostral ventral medulla, have been shown to provide rich array of neurochemical projections to the LC, including excitatory and inhibitory amino acids [111,112], corticotropin releasing factor (CRF) [113], adrenaline [114], and enkephalin (ENK) [111]. Anatomical studies using light and electron microscopy have revealed that the afferents to the noradrenergic dendrites in the LC demonstrate a topographic and monosynaptic pattern [115]. Electrophysiological recordings indicate that the activation of LC neurons is mediated by excitatory amino acid from PGi projections acting on non-NMDA receptors in the LC [116,117]. Approximately 73% of LC neurons exhibit an excitatory response by low-intensity stimulation of the PGi with a short latency of 11.3 msec, while only around 16% of the LC neurons showed inhibitory responses [117,118]. Notably, approximately 57% of the LC-projecting neurons in the PGi are enkephalinergic, implicating a strong modulatory component [111].

Functionally, PGi-LC projections play an important role in stress and memory-related behaviors. A recent study has found a functional PGi-related pathway—the nucleus of the solitary tract (NST) → PGi → LC → dorsal hippocampal CA1—that is involved in object recognition memory [119]. In response to a single resident-intruder exposure, both male and female rats with a short latency behavioral response exhibit remarkable c-Fos activation in PGi-LC ENK afferents, indicating the involvement of PGi-LC ENK afferents in modulating LC function. With repeated exposure, long-latency responses emerge, and PGi-LC ENK projections continue to be activated, suggesting sustained engagement of this pathway in social stress [120,121].

#### 3.1.2. Ventral Tegmental Area to LC

The VTA is a heterogeneous midbrain structure that has been reported to play diverse roles in behavioral and physiological functions, including the regulation of sleep/wakefulness, depression, reward prediction error, and addiction. It comprises multiple neuronal types, including ~65% dopamine, ~30% GABAergic, and ~5% glutamatergic neurons, and exhibits widespread projections throughout the brain [122,123]. Stimulation of VTA with kainic acid has been shown to increase the levels of the NE metabolite 3-methoxy-4-hydroxyphenolglycol in the prefrontal cortex, an effect that can be prevented by dorsal noradrenergic bundle knife cuts. This founding suggests that the effect of kainic acid-induced VTA stimulation influences the LC-derived dorsal bundle noradrenergic system [124]. More recent study has revealed that the VTA provides dense GABAergic projections to the central AMY, dorsal raphe nucleus (DRN), and LC. Utilizing electrophysiological recordings and optogenetic stimulation, it was found that the GABAergic neurons in VTA inhibited all recorded neurons of DRN but did not affect excitatory neurons in the AMY and LC [125]. However, due to the limited number of LC cell recordings, the existence of a functional GABAergic connection from the VTA to the LC was not confirmed. These observations suggest that further scientific inquiry is warranted to elucidate the functions of the VTA neuron projections to the LC.

#### 3.1.3. Suprachiasmatic Nucleus to LC

The suprachiasmatic nucleus (SCN) is a small hypothalamus region located above the optic chiasm and functions as the central circadian pacemaker, regulating daily rhythms, including the sleep–wake cycles [126]. Electrical stimulation of the LC produce an evoked potential in the SCN area, and reciprocally, stimulating the SCN area produce a similar potential recorded in the LC area, implying that a potential bidirectional communication may exists between the LC and SCN [127]. Study investigating the presynaptic inputs to LC-NE neurons has proved such directional projections from the SCN to the LC by using rabies-mediated trans-synaptic tracing techniques [1]. In addition to this direct input, retrograde trans-synaptic tracing has revealed several possible indirect pathways from the SCN to the LC also exist, involving the dorsomedial hypothalamic nucleus (DMH), paraventricular nucleus (PVN), and medial and ventrolateral pre-optic areas. Functional studies have shown that lesions of the DMH abolish circadian fluctuations in LC activity, underscoring the involvement of the SCN-DMH-LC circuit in regulating circadian and sleep-waking functions [128]. These findings suggest that SCN projection helps to shape LC activity across circadian and sleep–wake states, positioning the LC as a potential hub for relaying state-dependent information to support behaviors. However, further research is needed to clarify the mechanisms underlying this interaction.

#### 3.1.4. Periaqueductal Gray to LC

The midbrain periaqueductal gray (PAG) is a pivotal nucleus in the descending analgesia circuits. It mediates analgesic effects primarily through the activation of endogenous opioid acting on opioid receptors [129]. The LC contributes to endogenous descending pain control through its noradrenergic inputs to the SC [110] and, in turn, receives afferent inputs from the PAG [130]. A recent study has revealed that opioids can differentially modulate descending analgesia: they suppress descending analgesia through the PAG-LC pathway, while enhancing it through the PAG-RVM pathway [131]. Overall, these findings suggest that PAG-LC interaction represents a key descending pain control site, highlighting the LC’s role as a key integrator of analgesic signaling.

#### 3.1.5. Pontine and Cardiorespiratory Networks

The LC receives a large amount of input from pons nucleus that are central to autonomic homeostasis, forming part of a functional network for cardiorespiratory integration [1]. Hypothalamic regions, including the DMH and perifornical area (PeF), which constitute the central “Hypothalamic Defense Area” (HDA), have been shown to orchestrate cardiorespiratory responses to stress not through direct spinal projections, but via a sophisticated brainstem network [132]. Within this network, the pontine noradrenergic system—especially the A5 region and the LC—performs vital integrative and relay functions [133].

Anatomical and functional evidence has established that the A5 noradrenergic cell group and the LC are intricately interconnected within the brainstem noradrenergic network [132]. Both regions integrate inputs from major autonomic nuclei, including the DMH/PeF and the parabrachial nucleus (PB), and send extensive descending projections to key medullary cardiovascular centers—notably the rostral ventrolateral medulla (RVLM)—as well as to the intermediolateral cell column (IML) of the SC [95,133]. Through broad release of NE, these nuclei coordinate cardiovascular and respiratory outputs under stress conditions, thus modulating sympathetic tone and promoting cardiopulmonary homeostasis Kölliker-Fuse (KF) [132]. In parallel, the KF—a critical pontine respiratory rhythm generator—exhibits synchronized activity with the LC during stress-induced hyperventilation, further coupling breathing dynamics with autonomic arousal [131,132].Dysregulation of this circuit has been suggested to contribute to increased susceptibility to cardiovascular disorders. Collectively, these findings highlight the afferent from pontine nuclei to the LC as central components of the cardiorespiratory network, linking hypothalamic stress inputs to adaptive autonomic and respiratory outputs.

### 3.2. Cortical and Subcortical

#### 3.2.1. Amygdala to LC

The GABAergic neurons in the central amygdala (CeA) co-express CRF and project to the LC, modulating its neuronal activities [134,135,136]. CRF has been shown to increase the tonic firing rate of LC neurons from 1–2 Hz to 3–8 Hz while concurrently decreasing phasic firing, which results in anxiety-like behaviors and impaired attentional performance [137]. Studies using chemogenetics, optogenetics, and in vivo retrograde tracing techniques have demonstrated that elevated tonic firing of LC-NE neurons contributes to stress-related anxiety-like behaviors and aversion, whereas obstructing CRF receptors in the LC prevents these effects [138]. In a targeted approach, retrograde Cre viruses injected into the LC, combined with Cre-dependent AAV-DREADDs introduced into the CeA, selectively activated CeA neurons projecting to the LC [139]. This activation results in increased anxiety-like behaviors and leading to improved performance in memory tasks, accompanied by increased c-Fos expression in the LC region. Importantly, these effects were abolished by CRF1 receptor antagonist, confirming the role of CRF signaling in mediating the CeA-LC interaction [139]. Although research on the AMY-LC projection remains limited, current evidence suggests that CeA-derived CRF inputs to the LC critically shape stress-induced anxiety-like behaviors, which are likely achieved by shifting LC activity toward elevated tonic firing.

#### 3.2.2. Medial Prefrontal Cortex to LC

As discussed in Section 2, the mPFC is implicated in various physiological functions, including memory, pain modulation, goal-oriented behaviors, and impulse controls [140,141,142]. Notably, it is the only cortical region with direct projections to the LC [143]. The mPFC neurons establish direct excitatory projections to LC neurons while also providing inhibitory inputs to LC interneurons [143,144]. Recent investigations have found that inflammatory pain alters the activity of mPFC projections to the LC, impairing cognitive performance in male mice but not in females [145]. Interestingly, inhibition of mPFC projections to LC alleviates anxiety-like behavior in female mice. These findings underscore the role of mPFC-mediated top-down regulation of LC activity in cognition function and pain processing [145]. Moreover, this study reveals sex-dependent differences, with male LC neurons receiving more direct inputs from the mPFC, while females display greater connectivity between mPFC efferent and the LC as a whole.

#### 3.2.3. Paraventricular Nucleus to LC

By employing peroxidase labeling of axon terminals from the PVN and gold-silver staining of TH in LC dendrites, research has revealed that approximately 19% of terminals originating from PVN intersect with LC dendrites. Additionally, retrograde labeling and immunocytochemical analyses further illustrate that around 30% of LC-projecting PVN neurons are CRF-positive, while approximately 2% are ENK-positive [146]. Yawning, a behavior typically associated with arousal in the hypothalamus, has been employed to investigate this pathway. Upon stimulation of PVN neurons, a frequent yawning response is found in anesthetized, breathing rats, along with a significant increase c-Fos expression in CRF neurons within both the PVN and LC. These findings suggest the involvement of PVN-CRF projections to LC in arousal response during yawning [147].

## 4. The Modulation of Inputs to NE Neurons

**Figure 4:**
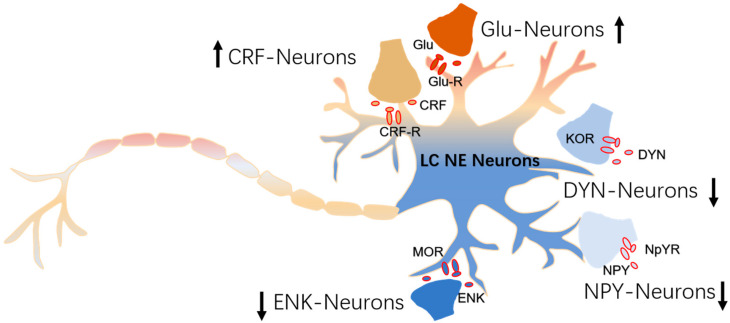
**The modulation of afferents to NE neurons.** Enkephalin (ENK) dynorphin (DYN) and neuropeptide Y (NPY) containing neurons inhibit the LC NE neurons, while corticotropin-releasing factor (CRF)- and glutamatergic (Glu)-containing neurons enhance their excitation. An upward arrow represents excitation, and a downward arrow represents inhibition. ENK, CRF, and Glu neurons modulate LC activity by releasing neurotransmitters or neuromodulators that directly act on postsynaptic receptors expressed on LC NE neurons. In contrast, DYN and NPY modulate LC functions via activation of presynaptic kappa-opioid receptors (KORs) and presynaptic neuropeptide Y receptors (NPYRs), thereby suppressing excitatory synaptic input.

LC NE neurons possess the capability to transition between tonic and phasic firing patterns, a feature fundamental to their regulation of various physiological functions. A key example of this regulation is cognitive performance, which improves with increasing LC activity, NE signaling, and arousal levels along an inverted-U shaped curve [148]. CRF exerted dose-dependent effects on distinct components of the task. Specifically, the highest dose (20 ng) enhanced reversal learning, while the lowest dose (2 ng) improved extradimensional set shifting. The dose–response relationship for extradimensional set shifting followed an inverted U-shaped pattern, with the highest dose showing no significant effect [149]. Afferent neurons influence these firing patterns via receptors or channels on the membrane of LC neurons. In this section, we will introduce and delineate how afferent neurons and their executive molecules modulate the excitation of LC NE neurons. The discharge rate and pattern of LC neurons are tuned by excitatory amino acids, CRF, and endogenous opioid afferents, which enable the LC to dynamically adjust its activity to suit different behavioral strategies appropriate to environmental demands [150]. Specifically, CRF was found to bias LC activity toward a higher tonic and lower phasic mode, a pattern associated with hyperarousal, reduced attention to ongoing behaviors, and increased vigilance to external stimuli [151]. Contrast, endogenous opioids, such as ENK, acting at the μ-opioid receptor (MOR), shifted LC activity toward the phasic pattern while reducing tonic pattern, thereby facilitating focused attention to ongoing behaviors [152]. Furthermore, dynorphin (DYN), acting via kappa-opioid receptor (KOR), exerts presynaptic inhibition on LC NE neurons, further contributing to the regulation of their excitability [150] (Figure 4).

### 4.1. CRF-Afferents

As described above, the CRF neurons originating from the PGi, PVN, and AMY project to LC NE neurons. CRF has been shown to increase tonic firing while decreasing phasic firing of LC NE neurons [153,154]. This effect is mediated by CRF binding to the CRF_1_ receptors, enhancing the Gs protein-coupled signaling pathway, leading to increased cAMP production [155]. Stress-induced alterations in LC activity are primarily mediated through this CRF–CRF1 signaling axis and can be prevented by blocking the CRF_1_ receptor. Sex differences have been observed in this pathway, which may underlie the higher prevalence of stress-related psychiatric disorders in women [35]. Interestingly, in males, CRF_1_ receptors exhibit increased β-arrestin2 binding, promoting CRF_1_ internalization and downregulation of signaling, whereas in female, CRF_1_ receptors demonstrate enhanced Gs protein coupling, resulting in greater activation of the cAMP signaling and heightened sensitivity to CRF [35].

### 4.2. ENK-Afferents

Opioid receptors, particularly MOR, are abundantly expressed in the LC. Projections from the PGi, PVN, and PAG deliver endogenous opioid peptides, including ENK, to LC NE neurons [47,66,146]. Opiates have been shown a decrease in the pacemaker activity of LC neurons by acute binding of opiates to the MOR [156,157].

Endogenous opioids bind to MORs in LC NE neurons, inhibiting their firing rate by increasing the conductance of G protein-coupled inwardly rectifying potassium (GIRK) channels [157,158] and suppressing a cAMP-regulated, sodium-dependent inward conductance [159,160,161]. Acute binding of opiates to the MOR also leads to decreased adenylyl cyclase (AC) activity and cAMP signaling [162]. However, chronic opiate administration restores both the firing rate and cAMP signaling to baseline by upregulating signaling proteins in the cAMP pathway, including AC1/8, cAMP-dependent protein kinase and cAMP-response element binding protein (CREB) [163]. Further studies also identify a crucial role for CREB in both the pacemaker activity and morphine-induced increase in LC firing rate using an LC slice culture model [164,165].

Desensitization of MORs is recognized as an initial phase in the development of opioid tolerance. Study has found that Orexin-A enhances the MOR desensitization in LC neurons of rats, potentially influencing opioid efficacy and adaptation [166]. The role of LC NE neurons function in chronic pain remains complex and somewhat controversial. Although LC-NE neurons exhibit acute endogenous analgesic properties via endogenous opioid pathways, evidence suggests that neuropathic injury could transform their analgesia function into one that perpetuates chronic pain [110]. Recent research using inhibitory optogenetics and conditional knockout approaches showed that MORs in LC neurons are required for normal nociception, and restoring either LC-MOR signaling or receptor expression reverses hypersensitivity in mice with spared nerve injury. Consequently, the destruction of MORs in LC neurons effectively transforms this analgesic function into a pain-prompting function [167].

### 4.3. DYN-Afferents

DYN-containing afferents directly target LC NE neurons by binding to KORs present in the axon terminals within the LC. Dual immunoelectron microscopy study has shown that many of these terminals also contain CRF or vesicular glutamate transporters, with approximately 35% of DYN terminals being immunoreactive for CRF, whereas only a small fraction of axon terminals contain both DYN and ENK, and these DYN/CRF afferents are primarily derived from the CeA [168,169,170]. In brain slice studies of the LC, activation of KORs by a highly selective agonist, CI-977, was found to depress excitatory synaptic potentials without affecting passive membrane properties or voltage-sensitive potassium currents of LC neurons [171]. Consistently, unlike MORs-mediated effects, in vivo study has shown that KOR agonists attenuate LC activation evoked by sciatic nerve stimulation without altering the spontaneous discharge of LC NE neurons [172]. These findings suggest that KORs may regulate LC NE neurons primarily by a presynaptic manner. During opiate withdrawal, the activation of LC neurons is markedly increased; however, this hyperactivation can be attenuated by microinfusing U50488, a selective κ-opioid agonist, into the LC, suggesting a presynaptic effect on glutamate release [165,173]. Collectively, these findings highlight that CeA-derived DYN afferents might provide a modulatory mechanism to fine-tune LC NE activity under both normal and pathological conditions.

### 4.4. Neuropeptide Y-Afferents

Neuropeptide Y (NPY) is a 36 amino-acid neuropeptide widely distributed throughout the nervous system. It plays a role in regulating various physiological and homeostatic processes, including stress, pain, and energy balance/appetitive behavior [174,175]. NPY binds to neuropeptide Y receptors (NPYR), a family of G protein-couped receptors (GPCR), of which seven subtypes (NPYR1-8) have been reported in vertebrates [175,176]. In mammals, five subtype (NPYR1, 2, 4, 5, and 6) are present, with four (NPYR1, 2, 4, and 5) are confirmed to be functional in humans [177]. These receptors are monomeric proteins belonging to the class A (rhodopsin-like) GPCR family [178]. Upon binding to ligands, it releases the Gi or G_0_ subunit of the heterotrimeric G protein complex, which subsequently inhibits adenylate cyclase activity and prevents ATP from being converted into the second messenger, cAMP [178]. Ultimately, cAMP levels are reduced, and the activities of calcium (Ca^2+)^ and potassium (K^+^) channels are modulated [179]. In rabbit smooth muscle cells, the NPYR2 and NPYR4 receptors have been shown to couple not only with Gs proteins but also interact with Gq proteins, thereby activating phospholipase C-βand promoting the production of inositol 1,4,5-trisphosphate [180]. In rat hypothalamic arcuate neurons, NPYR also regulated GIRK channels, in addition to K^+^ and Ca^2+^ channels [178].

It has been reported that NPY neurons in the arcuate nucleus (Arc) projected to the LC and colocalize with LC neurons expressing TH [181,182]. Using RNA scope and HCR3.0 in situ hybridization techniques, NPY expression was found to be restricted to TH^-^ LC neurons, which were identified as GABAergic neurons due to their co-expression of glutamate decarboxylase 1 [183]. Additionally, NPYR1, NPYR2 and NPYR5 were not detected in TH^+^ neurons of LC neurons [183].

The NPY system has garnered significant attention for its role in promoting stress resilience [184]. Recent studies suggest that the NPY signaling help reducing the risk of developing psychiatric disorders following traumatic events [185]. For example, veterans with high plasma NPY levels exhibited proactive coping strategies, while those with low NPY levels were more prone to PTSD symptoms [186,187]. Study detected transcript levels of NPY and NPYR in LC region from postmortem brain of control and suicide subjects, found that elevated NPYR1 levels were observed in the LC of male depressive patients, along with significantly increased NPY levels in both male and female patients [188]. Microinjected NPY (or a NPYR2 agonist) into vicinity of the LCV in rats has been shown to produce anxiolytic behavior, characterized by increased entries into the open arms of an elevated plus maze [189]. Pre-treating with NPY prior to single prolonged stress (SPS) exposure alleviated the stress-induced increase in TH expression [190]. Another study demonstrated that administering NPY immediately following SPS exposure prevented stress-induced reductions in NPYR2 receptor mRNA levels and increases in CRF_1_ receptors in the LC [191]. Collectively, these findings suggest that NPY afferents to the LC contribute to stress resilience and highlight the therapeutic potential of targeting the NPY system to mitigate stress-related disorders.

## 5. Conclusions and Future Direction

The LC-NE system stands as a central integrative hub in brain function, modulating diverse processes from arousal and cognition to emotional regulation and pain processing. Throughout this review, we have highlighted its evolutionary conservation, complex connectivity, and functional heterogeneity, the features that enable its profound influence on brain-wide states and behaviors. Despite significant advances, fundamental questions regarding how the LC’s diverse neuronal subpopulations, firing patterns, and circuit interactions collectively orchestrate such varied functions remain to be elucidated.

Therefore, caution should be warranted when we are attempting to construe LC-NE function, as it is not currently possible to provide a straightforward answer concerning its versatility. Even though the activation and inhibition of selected subsets of LC projections have been made easier with the advent of optogenetic and chemogenetic manipulation, our understanding between LC-NE activity and the behavior outcomes is still limited by its difficulty to accurately capture and reproduce the subtle dynamic changes in LC activity and its downstream effects.

When it comes to maladaptive behavior, research has revealed that dysregulated neuromodulation within this system—for instance, alterations in presynaptic terminal NE release dynamics or impairments in postsynaptic noradrenergic receptor signal transduction—is closely associated with the neurobiological mechanisms underlying psychiatric disorders such as anxiety, depression, cognitive impairment, ADHD, PTSD, and pain-related conditions. Previous studies primarily focused on the relationship between postsynaptic noradrenergic receptor dysfunction and psychiatric diseases. In recent years, advances in novel NE fluorescent probe technology have enabled real-time monitoring of NE dynamic release in mouse models and provided new possibilities for investigating the link between NE release and specific behavioral outcomes. Looking ahead, a primary challenge lies in deciphering the functional specialization of LC neurons based on their developmental origins, molecular profiles, and projection targets. The application of advanced tools, including cell-type-specific monitoring and manipulation, real-time NE sensors, and computational modeling, will be essential to resolve these subtleties and link LC dynamics to behavioral and cognitive outcomes

Translational efforts also have to be prioritized. The development of non-invasive biomarkers of LC integrity, perhaps through high-field neuroimaging or physiological correlates, offers promise for early diagnosis of neurodegenerative and neuropsychiatric disorders. Furthermore, neuromodulatory approaches such as targeted deep brain stimulation or transcranial magnetic stimulation, may allow selective intervention in LC-related circuits. Finally, cross-species studies integrating data from animal models and human investigations will be crucial to validate mechanistic insights and advance therapeutic innovations. By addressing these challenges, future research will not only illuminate the fundamental principles of neuromodulation but also pave the way for novel interventions for a range of brain disorders involving the LC-NE system.

## Figures and Tables

**Table 1:** The ratio between LC neurons and brain neurons.

Vertebrates	LC Neurons	Brain Neurons (×10^6^)	Ratio (×10^−6^)
Zebrafish	~10−20	~0.1	~100–200
Songbirds	~700	~2171 (Raven)	~0.32
Rats	~3000	~200	~15
Monkeys	~7000	~6391 (Rhesus)	~1.09
Humans	~15,000	~86,060	~0.17

Ratio = The number of LC cells/the number of brain cells.

## Data Availability

Data sharing is not applicable to this article as no new data were created or analyzed in this study.

## References

[1] Schwarz L.A., Miyamichi K., Gao X.J., Beier K.T., Weissbourd B., DeLoach K.E., Ren J., Ibanes S., Malenka R.C., Kremer E.J. (2015). Viral-genetic tracing of the input-output organization of a central noradrenaline circuit. Nature.

[2] Zerbi V., Floriou-Servou A., Markicevic M., Vermeiren Y., Sturman O., Privitera M., von Ziegler L., Ferrari K.D., Weber B., De Deyn P.P. (2019). Rapid Reconfiguration of the Functional Connectome after Chemogenetic Locus Coeruleus Activation. Neuron.

[3] Grimm C., Duss S.N., Privitera M., Munn B.R., Karalis N., Frässle S., Wilhelm M., Patriarchi T., Razansky D., Wenderoth N. (2024). Tonic and burst-like locus coeruleus stimulation distinctly shift network activity across the cortical hierarchy. Nat. Neurosci..

[4] Singh C., Oikonomou G., Prober D.A. (2015). Norepinephrine is required to promote wakefulness and for hypocretin-induced arousal in zebrafish. eLife.

[5] Maness E.B., Burk J.A., McKenna J.T., Schiffino F.L., Strecker R.E., McCoy J.G. (2022). Role of the locus coeruleus and basal forebrain in arousal and attention. Brain Res. Bull..

[6] Farrar M.J., Kolkman K.E., Fetcho J.R. (2018). Features of the structure, development, and activity of the zebrafish noradrenergic system explored in new CRISPR transgenic lines. J. Comp. Neurol..

[7] Holzschuh J., Ryu S., Aberger F., Driever W. (2001). Dopamine transporter expression distinguishes dopaminergic neurons from other catecholaminergic neurons in the developing zebrafish embryo. Mech. Dev..

[8] Castelino C.B., Schmidt M.F. (2010). What birdsong can teach us about the central noradrenergic system. J. Chem. Neuroanat..

[9] Sharma Y., Xu T., Graf W.M., Fobbs A., Sherwood C.C., Hof P.R., Allman J.M., Manaye K.F. (2010). Comparative anatomy of the locus coeruleus in humans and nonhuman primates. J. Comp. Neurol..

[10] Mouton P.R., Pakkenberg B., Gundersen H.J., Price D.L. (1994). Absolute number and size of pigmented locus coeruleus neurons in young and aged individuals. J. Chem. Neuroanat..

[11] Olkowicz S., Kocourek M., Lučan R.K., Porteš M., Fitch W.T., Herculano-Houzel S., Němec P. (2016). Birds have primate-like numbers of neurons in the forebrain. Proc. Natl. Acad. Sci. USA.

[12] Herculano-Houzel S., Lent R. (2005). Isotropic fractionator: A simple, rapid method for the quantification of total cell and neuron numbers in the brain. J. Neurosci..

[13] Ma P.M. (1994). Catecholaminergic systems in the zebrafish. I. Number, morphology, and histochemical characteristics of neurons in the locus coeruleus. J. Comp. Neurol..

[14] Jones B.E., Friedman L. (1983). Atlas of catecholamine perikarya, varicosities and pathways in the brainstem of the cat. J. Comp. Neurol..

[15] Tay T.L., Ronneberger O., Ryu S., Nitschke R., Driever W. (2011). Comprehensive catecholaminergic projectome analysis reveals single-neuron integration of zebrafish ascending and descending dopaminergic systems. Nat. Commun..

[16] Rink E., Wullimann M.F. (2004). Connections of the ventral telencephalon (subpallium) in the zebrafish (*Danio rerio*). Brain Res..

[17] Sánchez-Camacho C., Peña J.J., González A. (2003). Catecholaminergic innervation of the septum in the frog: A combined immunohistochemical and tract-tracing study. J. Comp. Neurol..

[18] Hoogland P.V. (1982). Brainstem afferents to the thalamus in a lizard, Varanus exanthematicus. J. Comp. Neurol..

[19] Bangma G.C., ten Donkelaar H. (1982). Afferent connections of the cerebellum in various types of reptiles. J. Comp. Neurol..

[20] Tuinhof R., Artero C., Fasolo A., Franzoni M.F., Ten Donkelaar H.J., Wismans P.G., Roubos E.W. (1994). Involvement of retinohypothalamic input, suprachiasmatic nucleus, magnocellular nucleus and locus coeruleus in control of melanotrope cells of Xenopus laevis: A retrograde and anterograde tracing study. Neuroscience.

[21] Barr H.J., Wall E.M., Woolley S.C. (2021). Dopamine in the songbird auditory cortex shapes auditory preference. Curr. Biol..

[22] Leutgeb S., Husband S., Riters L.V., Shimizu T., Bingman V.P. (1996). Telencephalic afferents to the caudolateral neostriatum of the pigeon. Brain Res..

[23] Bagnoli P., Burkhalter A. (1983). Organization of the afferent projections to the Wulst in the pigeon. J. Comp. Neurol..

[24] Rodman H.R., Karten H.J. (1995). Laminar distribution and sources of catecholaminergic input to the optic tectum of the pigeon (*Columbia livia*). J. Comp. Neurol..

[25] Takeuchi T., Duszkiewicz A.J., Sonneborn A., Spooner P.A., Yamasaki M., Watanabe M., Smith C.C., Fernández G., Deisseroth K., Greene R.W. (2016). Locus coeruleus and dopaminergic consolidation of everyday memory. Nature.

[26] Uematsu A., Tan B.Z., Ycu E.A., Cuevas J.S., Koivumaa J., Junyent F., Kremer E.J., Witten I.B., Deisseroth K., Johansen J.P. (2017). Modular organization of the brainstem noradrenaline system coordinates opposing learning states. Nat. Neurosci..

[27] Du W.J., Zhang R.W., Li J., Zhang B.B., Peng X.L., Cao S., Yuan J., Yuan C.D., Yu T., Du J.L. (2018). The Locus Coeruleus Modulates Intravenous General Anesthesia of Zebrafish via a Cooperative Mechanism. Cell Rep..

[28] Carter M.E., de Lecea L., Adamantidis A. (2013). Functional wiring of hypocretin and LC-NE neurons: Implications for arousal. Front. Behav. Neurosci..

[29] Carter M.E., Yizhar O., Chikahisa S., Nguyen H., Adamantidis A., Nishino S., Deisseroth K., de Lecea L. (2010). Tuning arousal with optogenetic modulation of locus coeruleus neurons. Nat. Neurosci..

[30] Poirier C., Boumans T., Vellema M., De Groof G., Charlier T.D., Verhoye M., Van der Linden A., Balthazart J. (2011). Own song selectivity in the songbird auditory pathway: Suppression by norepinephrine. PLoS ONE.

[31] Matsutani K., Tsuruoka M., Shinya A., Furuya R., Kawawa T. (2000). Stimulation of the locus coeruleus suppresses trigeminal sensorimotor function in the rat. Brain Res. Bull..

[32] Tsuruoka M., Willis W.D. (1996). Bilateral lesions in the area of the nucleus locus coeruleus affect the development of hyperalgesia during carrageenan-induced inflammation. Brain Res..

[33] Jones S.L., Gebhart G.F. (1986). Characterization of coeruleospinal inhibition of the nociceptive tail-flick reflex in the rat: Mediation by spinal alpha 2-adrenoceptors. Brain Res..

[34] West W.L., Yeomans D.C., Proudfit H.K. (1993). The function of noradrenergic neurons in mediating antinociception induced by electrical stimulation of the locus coeruleus in two different sources of Sprague-Dawley rats. Brain Res..

[35] Bangasser D.A., Wiersielis K.R., Khantsis S. (2016). Sex differences in the locus coeruleus-norepinephrine system and its regulation by stress. Brain Res..

[36] Pinos H., Collado P., Rodríguez-Zafra M., Rodríguez C., Segovia S., Guillamón A. (2001). The development of sex differences in the locus coeruleus of the rat. Brain Res. Bull..

[37] Garcia-Falgueras A., Pinos H., Collado P., Pasaro E., Fernandez R., Segovia S., Guillamon A. (2005). The expression of brain sexual dimorphism in artificial selection of rat strains. Brain Res..

[38] Garcia-Falgueras A., Pinos H., Fernández R., Collado P., Pasaro E., Segovia S., Guillamon A. (2006). Sexual dimorphism in hybrids rats. Brain Res..

[39] Busch C., Bohl J., Ohm T.G. (1997). Spatial, temporal and numeric analysis of Alzheimer changes in the nucleus coeruleus. Neurobiol. Aging.

[40] Ohm T.G., Busch C., Bohl J. (1997). Unbiased estimation of neuronal numbers in the human nucleus coeruleus during aging. Neurobiol. Aging.

[41] Mulvey B., Bhatti D.L., Gyawali S., Lake A.M., Kriaucionis S., Ford C.P., Bruchas M.R., Heintz N., Dougherty J.D. (2018). Molecular and Functional Sex Differences of Noradrenergic Neurons in the Mouse Locus Coeruleus. Cell Rep..

[42] Sun P., Wang J., Zhang M., Duan X., Wei Y., Xu F., Ma Y., Zhang Y.H. (2020). Sex-Related Differential Whole-Brain Input Atlas of Locus Coeruleus Noradrenaline Neurons. Front. Neural Circuits.

[43] Wang S., Wang Z., Mu Y. (2022). Locus Coeruleus in Non-Mammalian Vertebrates. Brain Sci..

[44] Borodovitsyna O., Flamini M., Chandler D. (2017). Noradrenergic Modulation of Cognition in Health and Disease. Neural Plast..

[45] Agster K.L., Clark B.D., Gao W.J., Shumsky J.S., Wang H.X., Berridge C.W., Waterhouse B.D. (2011). Experimental strategies for investigating psychostimulant drug actions and prefrontal cortical function in ADHD and related attention disorders. Anat. Rec..

[46] Hirschberg S., Li Y., Randall A., Kremer E.J., Pickering A.E. (2017). Functional dichotomy in spinal- vs prefrontal-projecting locus coeruleus modules splits descending noradrenergic analgesia from ascending aversion and anxiety in rats. eLife.

[47] Arnsten A.F. (2015). Stress weakens prefrontal networks: Molecular insults to higher cognition. Nat. Neurosci..

[48] Fan X., Song J., Ma C., Lv Y., Wang F., Ma L., Liu X. (2022). Noradrenergic signaling mediates cortical early tagging and storage of remote memory. Nat. Commun..

[49] Lapiz M.D., Morilak D.A. (2006). Noradrenergic modulation of cognitive function in rat medial prefrontal cortex as measured by attentional set shifting capability. Neuroscience.

[50] McGaughy J., Ross R.S., Eichenbaum H. (2008). Noradrenergic, but not cholinergic, deafferentation of prefrontal cortex impairs attentional set-shifting. Neuroscience.

[51] Spencer R.C., Berridge C.W. (2019). Receptor and circuit mechanisms underlying differential procognitive actions of psychostimulants. Neuropsychopharmacology.

[52] Arnsten A.F. (2007). Catecholamine and second messenger influences on prefrontal cortical networks of “representational knowledge”: A rational bridge between genetics and the symptoms of mental illness. Cereb. Cortex.

[53] Radley J.J., Rocher A.B., Janssen W.G., Hof P.R., McEwen B.S., Morrison J.H. (2005). Reversibility of apical dendritic retraction in the rat medial prefrontal cortex following repeated stress. Exp. Neurol..

[54] Loy R., Koziell D.A., Lindsey J.D., Moore R.Y. (1980). Noradrenergic innervation of the adult rat hippocampal formation. J. Comp. Neurol..

[55] Hansen N., Manahan-Vaughan D. (2015). Hippocampal long-term potentiation that is elicited by perforant path stimulation or that occurs in conjunction with spatial learning is tightly controlled by beta-adrenoreceptors and the locus coeruleus. Hippocampus.

[56] Amaral D.G., Foss J.A. (1975). Locus coeruleus lesions and learning. Science.

[57] Coradazzi M., Gulino R., Fieramosca F., Falzacappa L.V., Riggi M., Leanza G. (2016). Selective noradrenaline depletion impairs working memory and hippocampal neurogenesis. Neurobiol. Aging.

[58] Harley C. (1991). Noradrenergic and locus coeruleus modulation of the perforant path-evoked potential in rat dentate gyrus supports a role for the locus coeruleus in attentional and memorial processes. Prog. Brain Res..

[59] Lemon N., Aydin-Abidin S., Funke K., Manahan-Vaughan D. (2009). Locus coeruleus activation facilitates memory encoding and induces hippocampal LTD that depends on beta-adrenergic receptor activation. Cereb. Cortex.

[60] Devauges V., Sara S.J. (1991). Memory retrieval enhancement by locus coeruleus stimulation: Evidence for mediation by beta-receptors. Behav. Brain Res..

[61] Hansen N., Manahan-Vaughan D. (2015). Locus Coeruleus Stimulation Facilitates Long-Term Depression in the Dentate Gyrus That Requires Activation of β-Adrenergic Receptors. Cereb. Cortex.

[62] Babushkina N., Manahan-Vaughan D. (2022). Frequency-dependency of the involvement of dopamine D1/D5 and beta-adrenergic receptors in hippocampal LTD triggered by locus coeruleus stimulation. Hippocampus.

[63] Kempadoo K.A., Mosharov E.V., Choi S.J., Sulzer D., Kandel E.R. (2016). Dopamine release from the locus coeruleus to the dorsal hippocampus promotes spatial learning and memory. Proc. Natl. Acad. Sci. USA.

[64] Tsetsenis T., Badyna J.K., Li R., Dani J.A. (2022). Activation of a Locus Coeruleus to Dorsal Hippocampus Noradrenergic Circuit Facilitates Associative Learning. Front. Cell. Neurosci..

[65] Ul Haq R., Anderson M., Liotta A., Shafiq M., Sherkheli M.A., Heinemann U. (2016). Pretreatment with β-adrenergic receptor agonists facilitates induction of LTP and sharp wave ripple complexes in rodent hippocampus. Hippocampus.

[66] Lim E.P., Tan C.H., Jay T.M., Dawe G.S. (2010). Locus coeruleus stimulation and noradrenergic modulation of hippocampo-prefrontal cortex long-term potentiation. Int. J. Neuropsychopharmacol..

[67] Dahl M.J., Kulesza A., Werkle-Bergner M., Mather M. (2023). Declining locus coeruleus-dopaminergic and noradrenergic modulation of long-term memory in aging and Alzheimer’s disease. Neurosci. Biobehav. Rev..

[68] Kaouane N., Porte Y., Vallée M., Brayda-Bruno L., Mons N., Calandreau L., Marighetto A., Piazza P.V., Desmedt A. (2012). Glucocorticoids can induce PTSD-like memory impairments in mice. Science.

[69] McCall J.G., Siuda E.R., Bhatti D.L., Lawson L.A., McElligott Z.A., Stuber G.D., Bruchas M.R. (2017). Locus coeruleus to basolateral amygdala noradrenergic projections promote anxiety-like behavior. eLife.

[70] Fast C.D., McGann J.P. (2017). Amygdalar Gating of Early Sensory Processing through Interactions with Locus Coeruleus. J. Neurosci..

[71] Llorca-Torralba M., Suárez-Pereira I., Bravo L., Camarena-Delgado C., Garcia-Partida J.A., Mico J.A., Berrocoso E. (2019). Chemogenetic Silencing of the Locus Coeruleus-Basolateral Amygdala Pathway Abolishes Pain-Induced Anxiety and Enhanced Aversive Learning in Rats. Biol. Psychiatry.

[72] Wang Y., Pan Y., Cai Z., Lei C., Guo X., Cui D., Yuan Y., Lai B., Zheng P. (2021). Inputs from paraventricular nucleus of thalamus and locus coeruleus contribute to the activation of central nucleus of amygdala during context-induced retrieval of morphine withdrawal memory. Exp. Neurol..

[73] Haubrich J., Bernabo M., Nader K. (2020). Noradrenergic projections from the locus coeruleus to the amygdala constrain fear memory reconsolidation. eLife.

[74] Kromer L.F., Moore R.Y. (1980). A study of the organization of the locus coeruleus projections to the lateral geniculate nuclei in the albino rat. Neuroscience.

[75] Rogawski M.A., Aghajanian G.K. (1980). Modulation of lateral geniculate neurone excitability by noradrenaline microiontophoresis or locus coeruleus stimulation. Nature.

[76] Kayama Y., Negi T., Sugitani M., Iwama K. (1982). Effects of locus coeruleus stimulation on neuronal activities of dorsal lateral geniculate nucleus and perigeniculate reticular nucleus of the rat. Neuroscience.

[77] Rogawski M.A., Aghajanian G.K. (1980). Activation of lateral geniculate neurons by norepinephrine: Mediation by an alpha-adrenergic receptor. Brain Res..

[78] Ao Y., Yang B., Zhang C., Li S., Xu H. (2021). Application of quinpirole in the paraventricular thalamus facilitates emergence from isoflurane anesthesia in mice. Brain Behav..

[79] Kooiker C.L., Birnie M.T., Baram T.Z. (2021). The Paraventricular Thalamus: A Potential Sensor and Integrator of Emotionally Salient Early-Life Experiences. Front. Behav. Neurosci..

[80] Ao Y., Yang B., Zhang C., Wu B., Zhang X., Xing D., Xu H. (2021). Locus Coeruleus to Paraventricular Thalamus Projections Facilitate Emergence from Isoflurane Anesthesia in Mice. Front. Pharmacol..

[81] Beas B.S., Wright B.J., Skirzewski M., Leng Y., Hyun J.H., Koita O., Ringelberg N., Kwon H.B., Buonanno A., Penzo M.A. (2018). The locus coeruleus drives disinhibition in the midline thalamus via a dopaminergic mechanism. Nat. Neurosci..

[82] Arias-Carrión O., Stamelou M., Murillo-Rodríguez E., Menéndez-González M., Pöppel E. (2010). Dopaminergic reward system: A short integrative review. Int. Arch. Med..

[83] Isingrini E., Perret L., Rainer Q., Amilhon B., Guma E., Tanti A., Martin G., Robinson J., Moquin L., Marti F. (2016). Resilience to chronic stress is mediated by noradrenergic regulation of dopamine neurons. Nat. Neurosci..

[84] Zhang H., Chaudhury D., Nectow A.R., Friedman A.K., Zhang S., Juarez B., Liu H., Pfau M.L., Aleyasin H., Jiang C. (2019). α(1)- and β(3)-Adrenergic Receptor-Mediated Mesolimbic Homeostatic Plasticity Confers Resilience to Social Stress in Susceptible Mice. Biol. Psychiatry.

[85] Pradel K., Blasiak T., Solecki W.B. (2018). Adrenergic Receptor Agonists’ Modulation of Dopaminergic and Non-dopaminergic Neurons in the Ventral Tegmental Area. Neuroscience.

[86] Grenhoff J., Nisell M., Ferré S., Aston-Jones G., Svensson T.H. (1993). Noradrenergic modulation of midbrain dopamine cell firing elicited by stimulation of the locus coeruleus in the rat. J. Neural Transm. Gen. Sect..

[87] Paladini C.A., Williams J.T. (2004). Noradrenergic inhibition of midbrain dopamine neurons. J. Neurosci..

[88] Koob G.F., Volkow N.D. (2016). Neurobiology of addiction: A neurocircuitry analysis. Lancet Psychiatry.

[89] Solecki W.B., Kielbinski M., Wilczkowski M., Zajda K., Karwowska K., Joanna B., Rajfur Z., Przewłocki R. (2022). Regulation of cocaine seeking behavior by locus coeruleus noradrenergic activity in the ventral tegmental area is time- and contingency-dependent. Front. Neurosci..

[90] Jovanovic P., Wang Y., Vit J.P., Novinbakht E., Morones N., Hogg E., Tagliati M., Riera C.E. (2022). Sustained chemogenetic activation of locus coeruleus norepinephrine neurons promotes dopaminergic neuron survival in synucleinopathy. PLoS ONE.

[91] Parent M., Parent A. (2010). Substantia nigra and Parkinson’s disease: A brief history of their long and intimate relationship. Can. J. Neurol. Sci..

[92] Levey A.I., Qiu D., Zhao L., Hu W.T., Duong D.M., Higginbotham L., Dammer E.B., Seyfried N.T., Wingo T.S., Hales C.M. (2022). A phase II study repurposing atomoxetine for neuroprotection in mild cognitive impairment. Brain J. Neurol..

[93] Biondetti E., Gaurav R., Yahia-Cherif L., Mangone G., Pyatigorskaya N., Valabrègue R., Ewenczyk C., Hutchison M., François C., Arnulf I. (2020). Spatiotemporal changes in substantia nigra neuromelanin content in Parkinson’s disease. Brain.

[94] Giorgi F.S., Biagioni F., Galgani A., Pavese N., Lazzeri G., Fornai F. (2020). Locus Coeruleus Modulates Neuroinflammation in Parkinsonism and Dementia. Int. J. Mol. Sci..

[95] Bruinstroop E., Cano G., Vanderhorst V.G., Cavalcante J.C., Wirth J., Sena-Esteves M., Saper C.B. (2012). Spinal projections of the A5, A6 (locus coeruleus), and A7 noradrenergic cell groups in rats. J. Comp. Neurol..

[96] Nakajima K., Obata H., Iriuchijima N., Saito S. (2012). An increase in spinal cord noradrenaline is a major contributor to the antihyperalgesic effect of antidepressants after peripheral nerve injury in the rat. Pain.

[97] Pertovaara A., Wei H., Hämäläinen M.M. (1996). Lidocaine in the rostroventromedial medulla and the periaqueductal gray attenuates allodynia in neuropathic rats. Neurosci. Lett..

[98] Chen Q., Heinricher M.M. (2019). Plasticity in the Link between Pain-Transmitting and Pain-Modulating Systems in Acute and Persistent Inflammation. J. Neurosci..

[99] Kong D., Zhang Y., Gao P., Pan C., Deng H., Xu S., Tang D., Xiao J., Jiao Y., Yu W. (2023). The locus coeruleus input to the rostral ventromedial medulla mediates stress-induced colorectal visceral pain. Acta Neuropathol. Commun..

[100] Imbe H., Kimura A. (2016). Repeated forced swim stress affects the expression of pCREB and ΔFosB and the acetylation of histone H3 in the rostral ventromedial medulla and locus coeruleus. Brain Res. Bull..

[101] Aston-Jones G., Waterhouse B. (2016). Locus coeruleus: From global projection system to adaptive regulation of behavior. Brain Res..

[102] Cheun J.E., Yeh H.H. (1992). Modulation of GABAA receptor-activated current by norepinephrine in cerebellar Purkinje cells. Neuroscience.

[103] Cheun J.E., Yeh H.H. (1996). Noradrenergic potentiation of cerebellar Purkinje cell responses to GABA: Cyclic AMP as intracellular intermediary. Neuroscience.

[104] Moises H.C., Waterhouse B.D., Woodward D.J. (1981). Locus coeruleus stimulation potentiates Purkinje cell responses to afferent input: The climbing fiber system. Brain Res..

[105] Bickford P.C., Hoffer B.J., Freedman R. (1985). Interaction of norepinephrine with Purkinje cell responses to cerebellar afferent inputs in aged rats. Neurobiol. Aging.

[106] Stanley A.T., Post M.R., Lacefield C., Sulzer D., Miniaci M.C. (2023). Norepinephrine release in the cerebellum contributes to aversive learning. Nat. Commun..

[107] Llorca-Torralba M., Borges G., Neto F., Mico J.A., Berrocoso E. (2016). Noradrenergic Locus Coeruleus pathways in pain modulation. Neuroscience.

[108] Di Cesare Mannelli L., Micheli L., Crocetti L., Giovannoni M.P., Vergelli C., Ghelardini C. (2017). α2 Adrenoceptor: A Target for Neuropathic Pain Treatment. Mini Rev. Med. Chem..

[109] Li J., Wei Y., Zhou J., Zou H., Ma L., Liu C., Xiao Z., Liu X., Tan X., Yu T. (2022). Activation of locus coeruleus-spinal cordnoradrenergic neurons alleviates neuropathic pain in mice via reducing neuroinflammation from astrocytes and microglia in spinal dorsal horn. J. Neuroinflamm..

[110] Hickey L., Li Y., Fyson S.J., Watson T.C., Perrins R., Hewinson J., Teschemacher A.G., Furue H., Lumb B.M., Pickering A.E. (2014). Optoactivation of locus ceruleus neurons evokes bidirectional changes in thermal nociception in rats. J. Neurosci..

[111] Drolet G., Van Bockstaele E.J., Aston-Jones G. (1992). Robust enkephalin innervation of the locus coeruleus from the rostral medulla. J. Neurosci..

[112] Ennis M., Aston-Jones G. (1987). Two physiologically distinct populations of neurons in the ventrolateral medulla innervate the locus coeruleus. Brain Res..

[113] Valentino R.J., Page M., Van Bockstaele E., Aston-Jones G. (1992). Corticotropin-releasing factor innervation of the locus coeruleus region: Distribution of fibers and sources of input. Neuroscience.

[114] Pieribone V.A., Aston-Jones G., Bohn M.C. (1988). Adrenergic and non-adrenergic neurons in the C1 and C3 areas project to locus coeruleus: A fluorescent double labeling study. Neurosci. Lett..

[115] Van Bockstaele E.J., Colago E.E., Aicher S. (1998). Light and electron microscopic evidence for topographic and monosynaptic projections from neurons in the ventral medulla to noradrenergic dendrites in the rat locus coeruleus. Brain Res..

[116] Ennis M., Aston-Jones G., Shiekhattar R. (1992). Activation of locus coeruleus neurons by nucleus paragigantocellularis or noxious sensory stimulation is mediated by intracoerulear excitatory amino acid neurotransmission. Brain Res..

[117] Ennis M., Aston-Jones G. (1988). Activation of locus coeruleus from nucleus paragigantocellularis: A new excitatory amino acid pathway in brain. J. Neurosci..

[118] Aston-Jones G., Astier B., Ennis M. (1992). Inhibition of noradrenergic locus coeruleus neurons by C1 adrenergic cells in the rostral ventral medulla. Neuroscience.

[119] Mello-Carpes P.B., Izquierdo I. (2013). The Nucleus of the Solitary Tract → Nucleus Paragigantocellularis → Locus Coeruleus → CA1 region of dorsal hippocampus pathway is important for consolidation of object recognition memory. Neurobiol. Learn. Mem..

[120] Reyes B.A.S., Zhang X.Y., Dufourt E.C., Bhatnagar S., Valentino R.J., Van Bockstaele E.J. (2019). Neurochemically distinct circuitry regulates locus coeruleus activity during female social stress depending on coping style. Brain Struct. Funct..

[121] Reyes B.A., Zitnik G., Foster C., Van Bockstaele E.J., Valentino R.J. (2015). Social Stress Engages Neurochemically-Distinct Afferents to the Rat Locus Coeruleus Depending on Coping Strategy. eNeuro..

[122] Bouarab C., Thompson B., Polter A.M. (2019). VTA GABA Neurons at the Interface of Stress and Reward. Front. Neural Circuits.

[123] Margolis E.B., Toy B., Himmels P., Morales M., Fields H.L. (2012). Identification of rat ventral tegmental area GABAergic neurons. PLoS ONE.

[124] Deutch A.Y., Goldstein M., Roth R.H. (1986). Activation of the locus coeruleus induced by selective stimulation of the ventral tegmental area. Brain Res..

[125] Rahaman S.M., Chowdhury S., Mukai Y., Ono D., Yamaguchi H., Yamanaka A. (2022). Functional Interaction Between GABAergic Neurons in the Ventral Tegmental Area and Serotonergic Neurons in the Dorsal Raphe Nucleus. Front. Neurosci..

[126] Deurveilher S., Semba K. (2005). Indirect projections from the suprachiasmatic nucleus to major arousal-promoting cell groups in rat: Implications for the circadian control of behavioural state. Neuroscience.

[127] Legoratti-Sánchez M.O., Guevara-Guzmán R., Solano-Flores L.P. (1989). Electrophysiological evidences of a bidirectional communication between the locus coeruleus and the suprachiasmatic nucleus. Brain Res. Bull..

[128] Aston-Jones G., Chen S., Zhu Y., Oshinsky M.L. (2001). A neural circuit for circadian regulation of arousal. Nat. Neurosci..

[129] Lewis V.A., Gebhart G.F. (1977). Evaluation of the periaqueductal central gray (PAG) as a morphine-specific locus of action and examination of morphine-induced and stimulation-produced analgesia at coincident PAG loci. Brain Res..

[130] Lee H.S., Kim M.A., Waterhouse B.D. (2005). Retrograde double-labeling study of common afferent projections to the dorsal raphe and the nuclear core of the locus coeruleus in the rat. J. Comp. Neurol..

[131] Kim J.H., Gangadharan G., Byun J., Choi E.J., Lee C.J., Shin H.S. (2018). Yin-and-yang bifurcation of opioidergic circuits for descending analgesia at the midbrain of the mouse. Proc. Natl. Acad. Sci. USA.

[132] Carrillo-Franco L., González-García M., Morales-Luque C., Dawid-Milner M.S., López-González M.V. (2024). Hypothalamic Regulation of Cardiorespiratory Functions: Insights into the Dorsomedial and Perifornical Pathways. Biology.

[133] Rocha I., González-García M., Carrillo-Franco L., Dawid-Milner M.S., López-González M.V. (2024). Influence of Brainstem’s Area A5 on Sympathetic Outflow and Cardiorespiratory Dynamics. Biology.

[134] Van Bockstaele E.J., Chan J., Pickel V.M. (1996). Input from central nucleus of the amygdala efferents to pericoerulear dendrites, some of which contain tyrosine hydroxylase immunoreactivity. J. Neurosci. Res..

[135] Van Bockstaele E.J., Peoples J., Valentino R.J. (1999). Anatomic basis for differential regulation of the rostrolateral peri-locus coeruleus region by limbic afferents. Biol. Psychiatry.

[136] Cassell M.D., Freedman L.J., Shi C. (1999). The intrinsic organization of the central extended amygdala. Ann. N. Y. Acad. Sci..

[137] Redmond D.E., Huang Y.H. (1979). Current concepts. II. New Evid. A Locus Coeruleus-Norepinephrine Connect. anxiety. Life Sci..

[138] McCall J.G., Al-Hasani R., Siuda E.R., Hong D.Y., Norris A.J., Ford C.P., Bruchas M.R. (2015). CRH Engagement of the Locus Coeruleus Noradrenergic System Mediates Stress-Induced Anxiety. Neuron.

[139] Paretkar T., Dimitrov E. (2018). The Central Amygdala Corticotropin-releasing hormone (CRH) Neurons Modulation of Anxiety-like Behavior and Hippocampus-dependent Memory in Mice. Neuroscience.

[140] Ong W.Y., Stohler C.S., Herr D.R. (2019). Role of the Prefrontal Cortex in Pain Processing. Mol. Neurobiol..

[141] McKlveen J.M., Myers B., Herman J.P. (2015). The medial prefrontal cortex: Coordinator of autonomic, neuroendocrine and behavioural responses to stress. J. Neuroendocrinol..

[142] Van Eden C.G., Buijs R.M. (2000). Functional neuroanatomy of the prefrontal cortex: Autonomic interactions. Prog. Brain Res..

[143] Jodo E., Chiang C., Aston-Jones G. (1998). Potent excitatory influence of prefrontal cortex activity on noradrenergic locus coeruleus neurons. Neuroscience.

[144] Sara S.J., Hervé-Minvielle A. (1995). Inhibitory influence of frontal cortex on locus coeruleus neurons. Proc. Natl. Acad. Sci. USA.

[145] Cardenas A., Papadogiannis A., Dimitrov E. (2021). The role of medial prefrontal cortex projections to locus ceruleus in mediating the sex differences in behavior in mice with inflammatory pain. FASEB J..

[146] Reyes B.A., Valentino R.J., Xu G., Van Bockstaele E.J. (2005). Hypothalamic projections to locus coeruleus neurons in rat brain. Eur. J. Neurosci..

[147] Kita I., Seki Y., Nakatani Y., Fumoto M., Oguri M., Sato-Suzuki I., Arita H. (2006). Corticotropin-releasing factor neurons in the hypothalamic paraventricular nucleus are involved in arousal/yawning response of rats. Behav. Brain Res..

[148] Devilbiss D.M. (2019). Consequences of tuning network function by tonic and phasic locus coeruleus output and stress: Regulating detection and discrimination of peripheral stimuli. Brain Res..

[149] Snyder K., Wang W.W., Han R., McFadden K., Valentino R.J. (2012). Corticotropin-releasing factor in the norepinephrine nucleus, locus coeruleus, facilitates behavioral flexibility. Neuropsychopharmacology.

[150] Van Bockstaele E.J., Reyes B.A., Valentino R.J. (2010). The locus coeruleus: A key nucleus where stress and opioids intersect to mediate vulnerability to opiate abuse. Brain Res..

[151] Enman N.M., Reyes B.A.S., Shi Y., Valentino R.J., Van Bockstaele E.J. (2019). Sex differences in morphine-induced trafficking of mu-opioid and corticotropin-releasing factor receptors in locus coeruleus neurons. Brain Res..

[152] Downs A.M., McElligott Z.A. (2022). Noradrenergic circuits and signaling in substance use disorders. Neuropharmacology.

[153] Curtis A.L., Lechner S.M., Pavcovich L.A., Valentino R.J. (1997). Activation of the locus coeruleus noradrenergic system by intracoerulear microinfusion of corticotropin-releasing factor: Effects on discharge rate, cortical norepinephrine levels and cortical electroencephalographic activity. J. Pharmacol. Exp. Ther..

[154] Valentino R.J., Wehby R.G. (1988). Corticotropin-releasing factor: Evidence for a neurotransmitter role in the locus ceruleus during hemodynamic stress. Neuroendocrinology.

[155] Jedema H.P., Grace A.A. (2004). Corticotropin-releasing hormone directly activates noradrenergic neurons of the locus ceruleus recorded in vitro. J. Neurosci..

[156] Williams J.T., Egan T.M., North R.A. (1982). Enkephalin opens potassium channels on mammalian central neurones. Nature.

[157] Torrecilla M., Quillinan N., Williams J.T., Wickman K. (2008). Pre- and postsynaptic regulation of locus coeruleus neurons after chronic morphine treatment: A study of GIRK-knockout mice. Eur. J. Neurosci..

[158] Torrecilla M., Marker C.L., Cintora S.C., Stoffel M., Williams J.T., Wickman K. (2002). G-protein-gated potassium channels containing Kir3.2 and Kir3.3 subunits mediate the acute inhibitory effects of opioids on locus ceruleus neurons. J. Neurosci..

[159] Wang Y.Y., Aghajanian G.K. (1987). Excitation of locus coeruleus neurons by an adenosine 3′,5′-cyclic monophosphate-activated inward current: Extracellular and intracellular studies in rat brain slices. Synapse.

[160] Alreja M., Aghajanian G.K. (1993). Opiates suppress a resting sodium-dependent inward current and activate an outward potassium current in locus coeruleus neurons. J. Neurosci..

[161] Alreja M., Aghajanian G.K. (1994). QX-314 blocks the potassium but not the sodium-dependent component of the opiate response in locus coeruleus neurons. Brain Res..

[162] Duman R.S., Tallman J.F., Nestler E.J. (1988). Acute and chronic opiate-regulation of adenylate cyclase in brain: Specific effects in locus coeruleus. J. Pharmacol. Exp. Ther..

[163] Mazei-Robison M.S., Nestler E.J. (2012). Opiate-induced molecular and cellular plasticity of ventral tegmental area and locus coeruleus catecholamine neurons. Cold Spring Harb. Perspect. Med..

[164] Cao J.L., Vialou V.F., Lobo M.K., Robison A.J., Neve R.L., Cooper D.C., Nestler E.J., Han M.H. (2010). Essential role of the cAMP-cAMP response-element binding protein pathway in opiate-induced homeostatic adaptations of locus coeruleus neurons. Proc. Natl. Acad. Sci. USA.

[165] Han M.H., Bolaños C.A., Green T.A., Olson V.G., Neve R.L., Liu R.J., Aghajanian G.K., Nestler E.J. (2006). Role of cAMP response element-binding protein in the rat locus ceruleus: Regulation of neuronal activity and opiate withdrawal behaviors. J. Neurosci..

[166] Mohammad Ahmadi Soleimani S., Azizi H., Pachenari N., Mirnajafi-Zadeh J., Semnanian S. (2017). Enhancement of μ-opioid receptor desensitization by orexin-A in rat locus coeruleus neurons. Neuropeptides.

[167] Norris M.R.K., Kuo C.C., Kim J.R., Dunn S.S., Borges G., Thang L.V., McCall J.G. (2023). Endogenous opioids gate the locus coeruleus pain generator. bioRxiv Prepr. Serv. Biol..

[168] Reyes B.A., Johnson A.D., Glaser J.D., Commons K.G., Van Bockstaele E.J. (2007). Dynorphin-containing axons directly innervate noradrenergic neurons in the rat nucleus locus coeruleus. Neuroscience.

[169] Reyes B.A., Drolet G., Van Bockstaele E.J. (2008). Dynorphin and stress-related peptides in rat locus coeruleus: Contribution of amygdalar efferents. J. Comp. Neurol..

[170] Barr J., Van Bockstaele E.J. (2005). Vesicular glutamate transporter-1 colocalizes with endogenous opioid peptides in axon terminals of the rat locus coeruleus. Anat. record. Part A Discov. Mol. Cell. Evol. Biol..

[171] Pinnock R.D. (1992). A highly selective kappa-opioid receptor agonist, CI-977, reduces excitatory synaptic potentials in the rat locus coeruleus in vitro. Neuroscience.

[172] Kreibich A., Reyes B.A., Curtis A.L., Ecke L., Chavkin C., Van Bockstaele E.J., Valentino R.J. (2008). Presynaptic inhibition of diverse afferents to the locus ceruleus by kappa-opiate receptors: A novel mechanism for regulating the central norepinephrine system. J. Neurosci..

[173] Redmond D.E., Huang Y.H. (1982). The primate locus coeruleus and effects of clonidine on opiate withdrawal. J. Clin. Psychiatry.

[174] Allen Y.S., Adrian T.E., Allen J.M., Tatemoto K., Crow T.J., Bloom S.R., Polak J.M. (1983). Neuropeptide Y distribution in the rat brain. Science.

[175] Yi M., Li H., Wu Z., Yan J., Liu Q., Ou C., Chen M. (2018). A Promising Therapeutic Target for Metabolic Diseases: Neuropeptide Y Receptors in Humans. Cell. Physiol. Biochem..

[176] Larhammar D., Salaneck E. (2004). Molecular evolution of NPY receptor subtypes. Neuropeptides.

[177] Pedragosa-Badia X., Stichel J., Beck-Sickinger A.G. (2013). Neuropeptide Y receptors: How to get subtype selectivity. Front. Endocrinol..

[178] Michel M.C., Beck-Sickinger A., Cox H., Doods H.N., Herzog H., Larhammar D., Quirion R., Schwartz T., Westfall T. (1998). XVI. International Union of Pharmacology recommendations for the nomenclature of neuropeptide Y, peptide YY, and pancreatic polypeptide receptors. Pharmacol. Rev..

[179] Cabrele C., Beck-Sickinger A.G. (2000). Molecular characterization of the ligand-receptor interaction of the neuropeptide Y family. J. Pept. Sci. Off. Publ. Eur. Pept. Soc..

[180] Misra S., Murthy K.S., Zhou H., Grider J.R. (2004). Coexpression of Y1, Y2, and Y4 receptors in smooth muscle coupled to distinct signaling pathways. J. Pharmacol. Exp. Ther..

[181] Everitt B.J., Hökfelt T., Terenius L., Tatemoto K., Mutt V., Goldstein M. (1984). Differential co-existence of neuropeptide Y (NPY)-like immunoreactivity with catecholamines in the central nervous system of the rat. Neuroscience.

[182] Yoon Y.S., Lee J.S., Lee H.S. (2013). Retrograde study of CART- or NPY-neuronal projection from the hypothalamic arcuate nucleus to the dorsal raphe and/or the locus coeruleus in the rat. Brain Res..

[183] Caramia M., Romanov R.A., Sideromenos S., Hevesi Z., Zhao M., Krasniakova M., Xu Z.D., Harkany T., Hökfelt T.G.M. (2023). Neuronal diversity of neuropeptide signaling, including galanin, in the mouse locus coeruleus. Proc. Natl. Acad. Sci. USA.

[184] Zitnik G.A. (2016). Control of arousal through neuropeptide afferents of the locus coeruleus. Brain Res..

[185] Enman N.M., Sabban E.L., McGonigle P., Van Bockstaele E.J. (2015). Targeting the Neuropeptide Y System in Stress-related Psychiatric Disorders. Neurobiol. Stress.

[186] Sah R., Ekhator N.N., Jefferson-Wilson L., Horn P.S., Geracioti T.D. (2014). Cerebrospinal fluid neuropeptide Y in combat veterans with and without posttraumatic stress disorder. Psychoneuroendocrinology.

[187] Yehuda R., Brand S., Yang R.K. (2006). Plasma neuropeptide Y concentrations in combat exposed veterans: Relationship to trauma exposure, recovery from PTSD, and coping. Biol. Psychiatry.

[188] Barde S., Aguila J., Zhong W., Solarz A., Mei I., Prud’homme J., Palkovits M., Turecki G., Mulder J., Uhlén M. (2024). NPY, CCK and their receptors in five brain regions in major depressive disorder with transcriptomic analysis of locus coeruleus neurons. Eur. Neuropsychopharmacol..

[189] Kask A., Rägo L., Harro J. (1998). Anxiolytic-like effect of neuropeptide Y (NPY) and NPY13-36 microinjected into vicinity of locus coeruleus in rats. Brain Res..

[190] Serova L.I., Tillinger A., Alaluf L.G., Laukova M., Keegan K., Sabban E.L. (2013). Single intranasal neuropeptide Y infusion attenuates development of PTSD-like symptoms to traumatic stress in rats. Neuroscience.

[191] Sabban E.L., Laukova M., Alaluf L.G., Olsson E., Serova L.I. (2015). Locus coeruleus response to single-prolonged stress and early intervention with intranasal neuropeptide Y. J. Neurochem..

